# The unstable concept of “stability” in osteoporotic 2-part proximal humerus fractures

**DOI:** 10.2340/17453674.2025.44266

**Published:** 2025-07-23

**Authors:** Stig BRORSON

**Affiliations:** Centre for Evidence-Based Orthopaedics, Zealand University Hospital, Køge, and Department of Clinical Medicine, University of Copenhagen, Denmark

For an engineer, the concept of stability has a distinct meaning. Lack of stability can lead to the collapse of bridges and cranes. However, to what extent does the engineering term apply to fracture stability in the most common pattern of displaced proximal humerus fractures, and does it provide a reliable guide in clinical decision-making?

Stability considerations for proximal humerus fractures are commonly found in clinical and scientific contexts. They can appear as truisms like:

*Whereas stable fractures are generally and successfully treated by closed means, the majority of unstable and displaced fractures require surgical treatment* [1].

or

*While most of these injuries are treated non-operatively with satisfactory results, surgical intervention is used for the more unstable fractures* [2].

When a fracture is considered to need surgical intervention, it is classified as “unstable.” However, there is limited experience with patients who are suitable for surgery but have an “unstable” 2-part fracture that has been treated non-surgically. As the concept of stability is often used to justify the need for surgery, it is important to examine the concept and its application more closely.

## Definitions of stability

Following the “Arbeitsgemeinschaft für Osteosynthesefragen” (AO):

*A stable fracture is defined as a fracture that does not visibly displace under physiological load* [3].

Load and visible displacement seem to be the key to understanding the concept of fracture stability. However, does the same principle apply to a broken proximal femur and a broken proximal humerus? While the load is a concern in injuries involving weightbearing bones and joints, what “physiological load” of the proximal humerus means in an elderly person becomes more blurred. Whether the need for surgical reconstruction or joint replacement of the proximal humerus can be inferred from the assessment of visible displacement from imaging needs to be justified.

Many 2-part fractures are inherently unstable, in the sense that the deforming forces exerted by the deltoid and pectoralis major will counter the anatomical apposition of the shaft with the humeral head.

Ideally, defining a scientific concept involves determining necessary and sufficient conditions for the use of the concept. We should distinguish stability, which is an inherent property of a construct, and the surgeon’s concept of stability, which has been formed by multiple modifying factors like the prevalent biomechanical knowledge, training, local traditions, recommendations by implant providers, and personal preferences (e.g., “platers” vs “nailers”). Concepts are shaped within the orthopedic community and change over time. Some decisions, such as determining the need for surgery, are adopted through pattern recognition during our training as orthopedic surgeons and through our personal clinical experience.

Even if the AO definition is interpreted in purely mechanical terms, its application provides insufficient guidance in clinical decision-making.

## Fracture morphology as a predictor of instability

Morphological patterns on images are commonly interpreted in terms of stability. When determining the stability of an isolated Weber Type B ankle fracture, we look at fibular displacement as an indirect measure of the state of the medial ligaments, attempting to predict the stability of the fracture [4]. Similar pattern recognition by indirect measures of stability can be found in the assessment of proximal humerus fractures.

**Figure 1:**
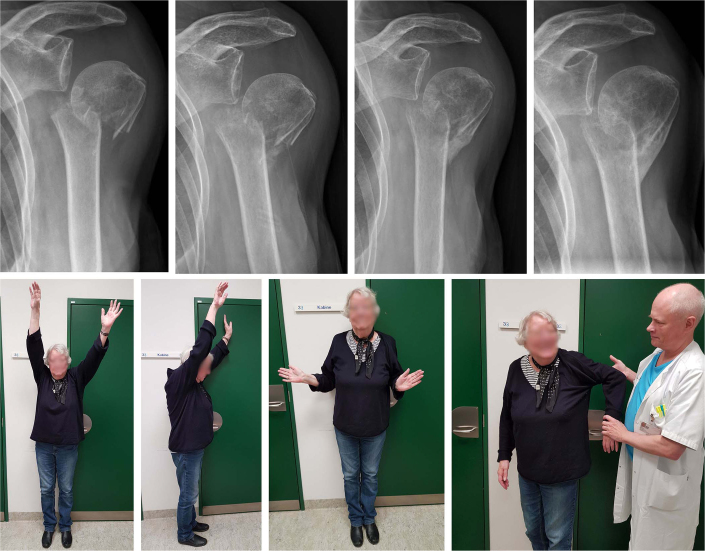
A 79-year-old healthy female with a 2-part surgical neck fracture, accompanied by medial translation of the humeral shaft. A lateral bone bridge was formed, allowing the patient to regain pain-free shoulder function. However, external rotation remained restricted. Radiographs at admission, 6 weeks, 12 weeks, and 24 weeks. Clinical photos at 24 weeks.

Two-part surgical neck fractures can be further divided according to the translation of the humeral shaft or by varus displacement. If there is no medial (calcar) support, the fracture is often considered unstable and in need of surgical fixation (Figure 1). These fracture patterns are dealt with in the most popular platform for young surgeons, the AO Surgery Reference [5].

Simple surgical neck fractures with angulation are:

*… most often unstable even though no comminution is present* [5].

Non-impacted 2-part surgical neck fractures with medial translation are considered unstable and in need of surgery (Figure 1):

*Because of the instability of these fractures, surgical fixation is often recommended* [5].

A threshold for instability is given:

*Signs of instability are a translation of the humeral shaft of more than 33%*.

Interestingly, osteoporotic bone serves as a supporting indication for surgery [5].

However, medially translated fractures often have a good clinical outcome in the elderly despite being deemed unstable and leading to malunion (see Figure 1).

**Figure 2:**
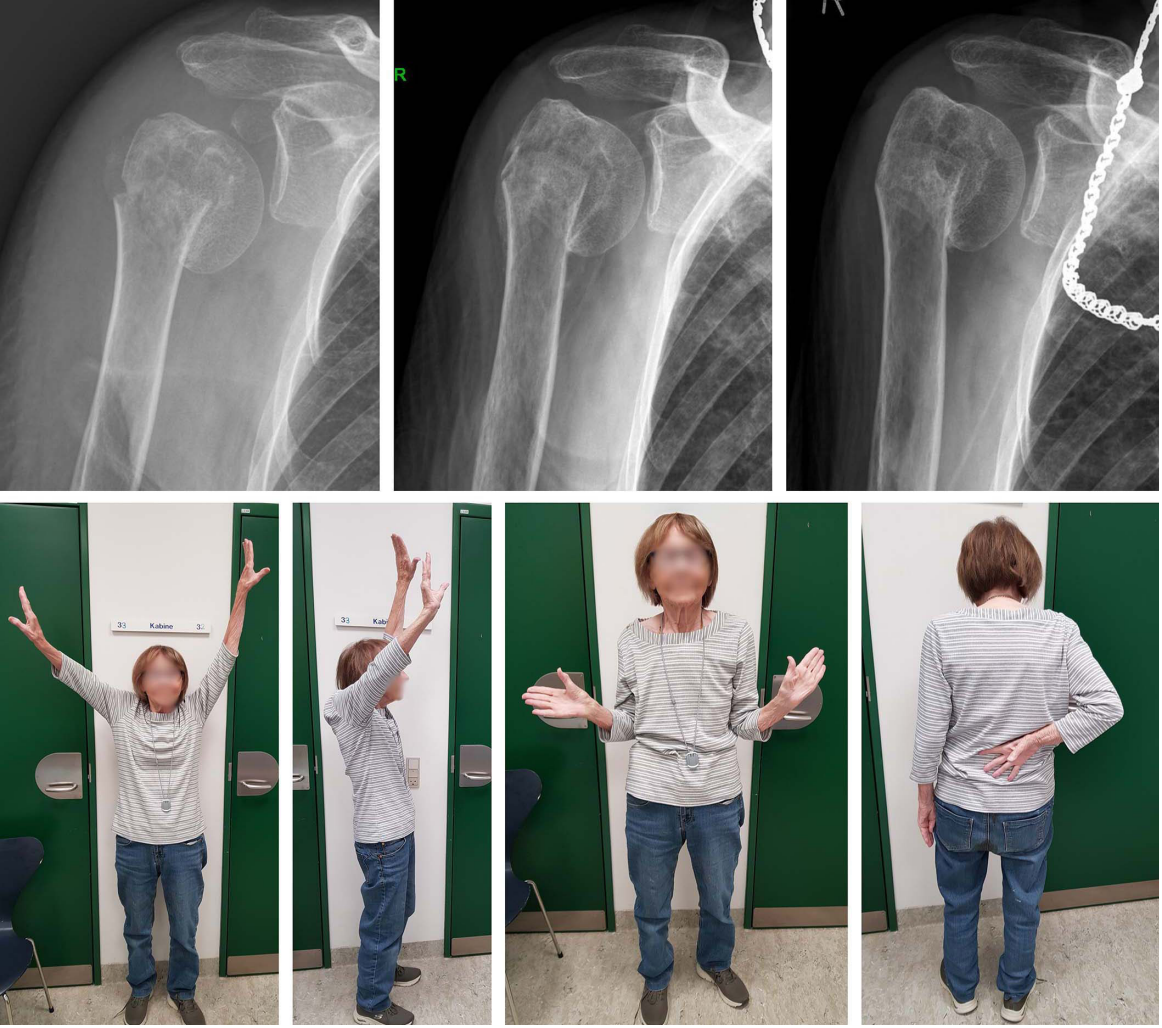
An 89-year-old nursing home resident with a varus displaced 2-part surgical neck fracture. The fracture healed in varus malunion, but the patient obtained pain-free shoulder function. Radiographs at admission, 6 weeks, and 24 weeks. Clinical photos at 24 weeks.

**Figure 3:**
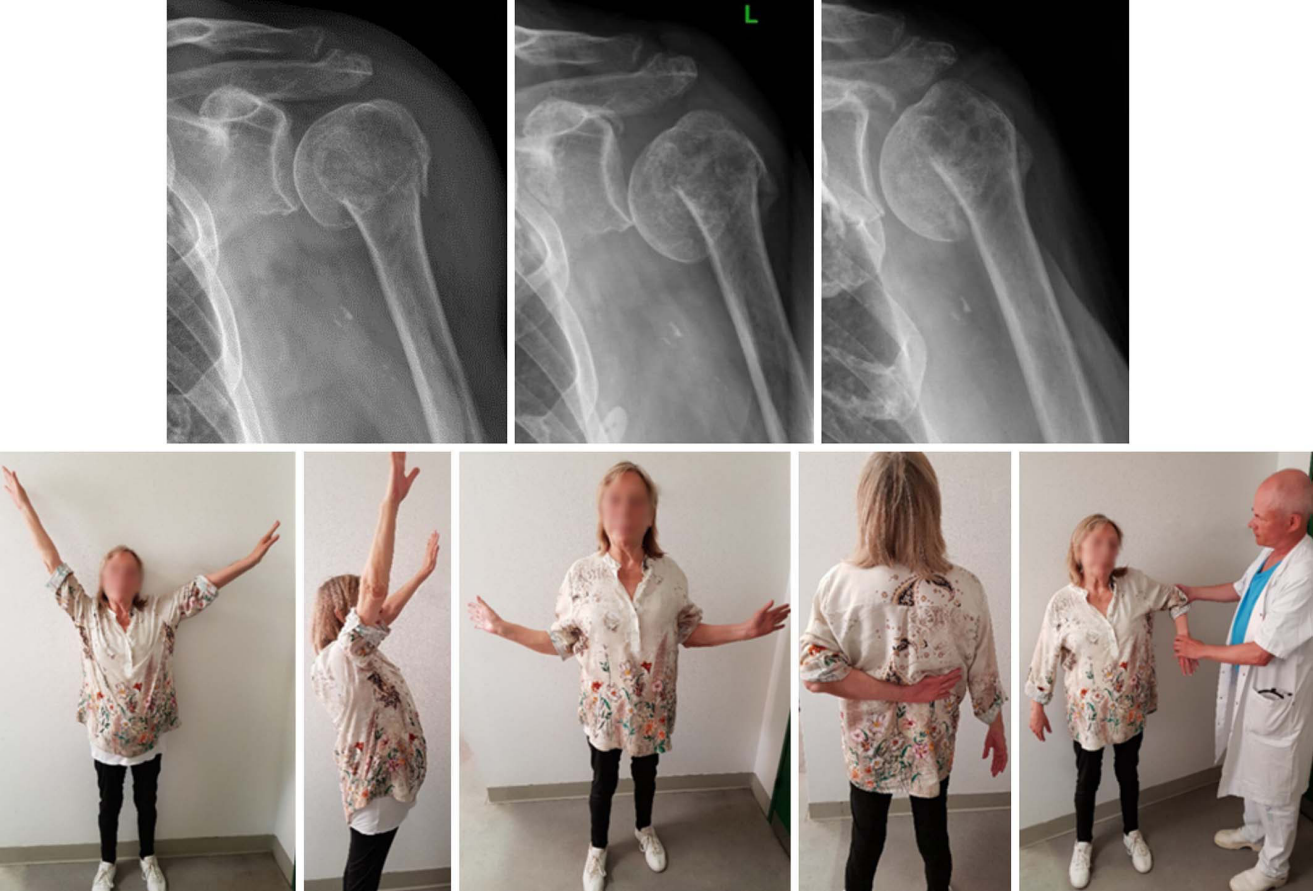
A 71-year-old female with a 2-part surgical neck fracture. The fracture collapsed into varus within the first week but eventually healed resulting in pain-free shoulder function. Radiographs at admission, 1 week, and 12 weeks. Clinical photos at 12 weeks.

**Figure 4:**
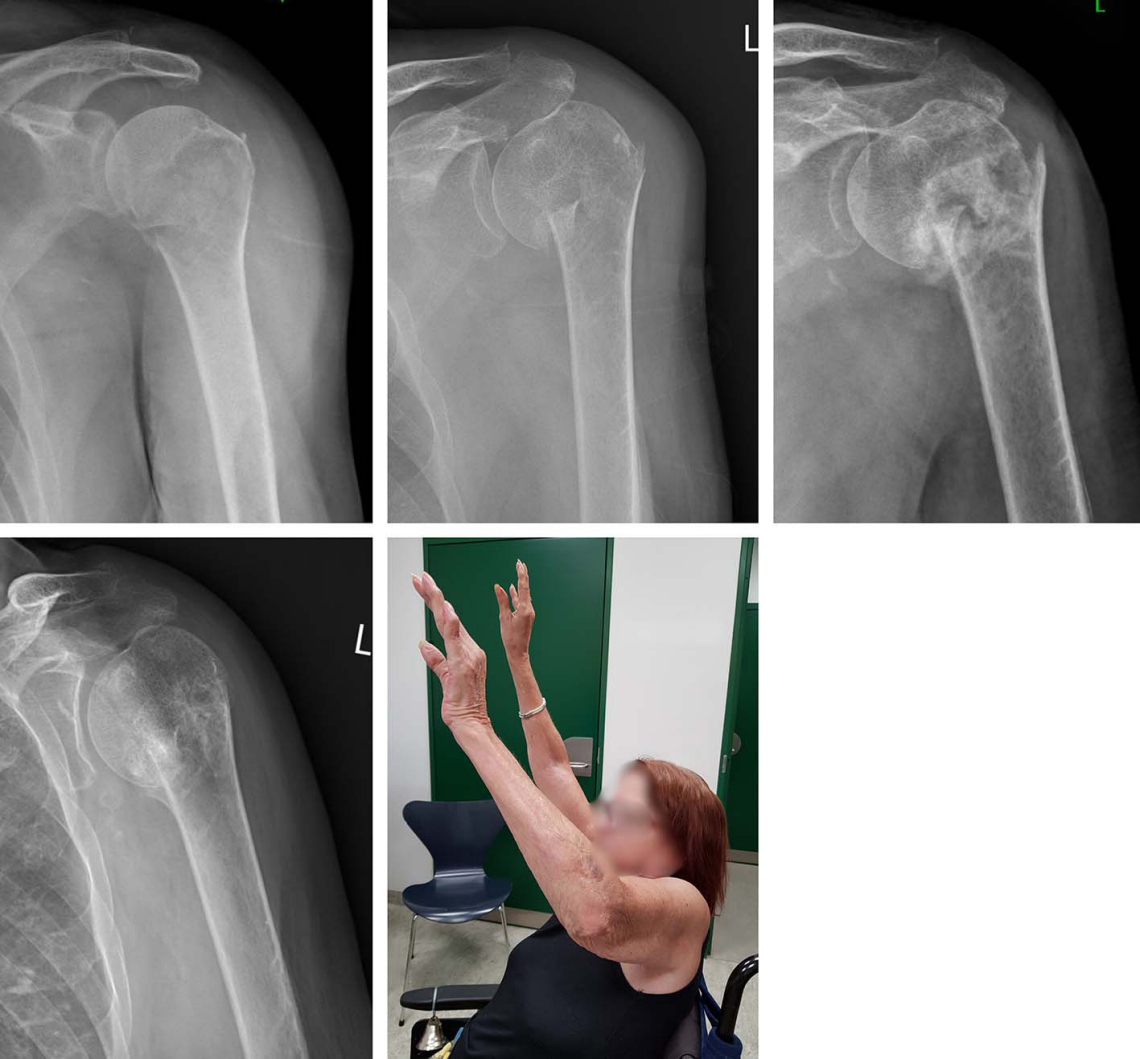
A progressive varus collapse of a minimally displaced fracture in a disabled nursing home resident. Despite the collapse and partial resorption of the humeral head, the pre-injury function was restored. Radiographs at admission, 2 weeks, 6 weeks, and 24 weeks. Clinical photo at 24 weeks.

Varus displaced 2-part surgical neck fractures can appear as primary varus displaced (Figure 2), as secondary to humeral head collapse (Figure 3), or as a complication to initially minimally displaced fractures (Figure 4). These patterns also have good potential for natural healing as illustrated by the patient cases.

The recommendations for surgical treatment of displaced 2-part surgical neck fractures, withheld on didactic platforms, textbooks, and technical guides, do not align with conclusions drawn from randomized trials and prospective cohort studies on 2-part fractures reporting no benefits from surgery [6-9].

## Instability derived from changes in fracture morphology

Most shoulder fractures change their morphology over time, which may lead assessors of imaging to believe there is instability. However, these changes are expected, especially in osteoporotic bone, and do not reliably correlate with patient outcomes. Morphological changes, including varus collapse, will often proceed regardless of surgical fixation. Attempts to achieve stability using angle-stable implants are often unsuccessful, even when multiple locking screws are inserted into the humeral head as stability is not achieved. The fragile osteoporotic humeral bone cannot withstand the stiff implant and loss of reduction or cut-out into the joint often follows.

Our inclination toward stabilizing osteoporotic proximal humerus fractures may be biomechanical meaningful, but surgical fixation has not been demonstrated to perform better than non-surgical treatment [8]. On the contrary, high failure rates have been reported, even from renowned institutions [10]. Our intuitions and expectations as to what will happen are not always supported by the best evidence and seem to underestimate the natural course of bone healing. Additionally, the trend to surgically fix fractures of the proximal humerus in the elderly may introduce cognitive bias, especially if we rely solely on observations from patients deemed unsuitable for surgery when making clinical decisions.

“Instability” of osteoporotic proximal humerus fractures is not an independent indication for surgical fixation

*In conclusion,* 2-part surgical neck fractures displace during the early healing phase, often helpfully. If the term “instability” is used interchangeably with (inevitable and favorable) displacements, and if “instability” is then used to define the indication for operative intervention, too many patients will undergo unnecessary surgical treatment.

As orthopedic surgeons and evidence-based practitioners, we should acknowledge the healing power of nature, *vis medicatrix naturae* (attributed to Hippocrates), or the natural course, when considering elderly patients with 2-part proximal humerus fractures for surgery. We should weigh the risk of causing harm to our patients through surgery against our inclination towards reduction and surgical fixation in anatomical position.

Imaging-based clinical decisions based on surgeons’ intuitions on “stability”’ do not provide a reliable guide to evidence-based and patient-centered care and may result in unnecessary surgeries.

## Notes

Complete disclosure of interest form according to ICMJE is available on the article page, doi: 10.2340/17453674.2025.44266

Handling co-editors: Taco Gosens

*Acta* thanks Simon Lambert and Olof Wolf for help with peer review of this manuscript.
